# Standardized subxiphoid echocardiography in swine: procedural guide and reference values

**DOI:** 10.3389/fcvm.2025.1709049

**Published:** 2025-11-13

**Authors:** Sebastian Billig, Matthias Derwall, Moritz Uhlig, Rachad Zayat, Sergej Yelenski

**Affiliations:** 1 Department of Anesthesiology, Faculty of Medicine, RWTH Aachen University, Aachen, Germany; 2 Department of Anesthesia, Critical Care and Pain Medicine, St. Johannes Hospital, Dortmund, Germany; 3 Department of Cardiac Surgery, Faculty of Medicine, RWTH Aachen University, Aachen, Germany; 4 Department of Thoracic Surgery, Faculty of Medicine, RWTH Aachen University, Aachen, Germany

**Keywords:** echocardiography, swine, translational research, subxiphoid echocardiography, speckle tracking

## Abstract

**Background:**

Subxiphoid echocardiography (SE) via small surgical access offers a minimally invasive imaging technique for preclinical swine models. However, a detailed methodological description that includes the surgical approach and imaging planes is lacking. The aim of this study was to standardize SE performed via a subxiphoid surgical approach and to provide reference values for future research.

**Methods:**

SE was performed in 19 female German Landrace pigs under general anesthesia using a defined imaging protocol. After the induction of general anesthesia, a small subxiphoid incision was made to optimize the image quality for SE. The echocardiographic imaging protocol was adapted from human 2D-transthoracic echocardiography and included apical 2-, 3-, and 5-chamber views and Doppler measurements of blood flow and tissue velocities. Furthermore, speckle tracking was used to assess left ventricular (LV) and right ventricular (RV) myocardial deformation.

**Results:**

During SE, the animals were under stable anesthesia with a heart rate of 65 ± 12 beats per minute, and a mean arterial pressure of 88 ± 12 mmHg. Blood gas values were within physiological ranges. High-quality images of both ventricles were consistently achieved using the established imaging protocol. One animal showed a severe aortic stenosis and was excluded from the analysis. The biplane LV stroke volume was 45 ± 13 ml and the LV ejection fraction was 67% ± 11%. LV-global longitudinal strain (GLS) was −22.4% ± 3.3%, and RV free wall longitudinal strain was −23.7% ± 5%. The V_max_ of the aortic valve was 1.6 ± 0.3 m/s.

**Conclusion:**

SE offers a standardized and reproducible imaging technique for the assessment of high-quality apical views in swine models. This study provides procedural guidance and reference values for translational research applications.

## 1 Introduction

Swine are widely used in preclinical research because of their anatomical and physiological similarities to humans (1–3). Their heart size and hemodynamic properties closely resemble those of human patients, making pigs a valuable model for translational research (4–6). Owing to its ease of application and ability to perform real-time imaging, echocardiography constitutes a key modality in this research. However, direct application of human echocardiography to pigs is not possible (7). A careful adoption of the technique is needed to account for species-specific anatomical differences (8–10): In contrast to an oblique heart axis in humans, the posture of pigs and the keel-shaped thorax results in a perpendicular orientation of the heart relative to the body axis (4). Additionally, the swine heart shows a counterclockwise rotation compared with the orientation of the human heart, with a more cranially situated right ventricle (RV) and a more caudally situated left ventricle (LV) (4, 11). As a result of these anatomical differences, the acquisition of standardized echocardiographic views in pigs is not feasible in the same way as it is in humans. Furthermore, the narrow and stable rib cage limits adequate acoustic coupling. Apical views, which are critical for accurate functional evaluation and Doppler assessment, are particularly difficult to obtain in intubated swine during anesthesia (12–14). This is a significant limitation for conventional transthoracic echocardiography (TTE), since most of the swine examined in preclinical research will be intubated. Therefore, TTE is insufficient for a comprehensive echocardiographic evaluation in swine. Transesophageal echocardiography (TOE) may represent a viable alternative, especially when transthoracic access is restricted by surgical procedures. However, TOE requires experienced sonographers and sophisticated equipment, which is not universally available. Additionally, the anatomic position of the left main bronchus between the right atrium and the esophagus can hinder right ventricular imaging via TOE (8, 9). Intracardiac echocardiography (ICE) offers direct visualization of cardiac structures but is highly invasive and technically challenging (15). Nevertheless, like TOE, ICE demands advanced expertise and high-end sonographic equipment (16).

Subxiphoid echocardiography (SE) via a small surgical incision caudal to the xiphoid process offers an alternative approach that is easier, less resource intensive, and readily adaptable for personnel already trained in TTE (17–19). This method allows direct contact between the ultrasound probe and the pericardium, minimizing interference from the lungs, ribs and airways. SE therefore facilitates high-quality imaging of LV and RV apical views. Although SE has been described in various experimental contexts, a standardized protocol and reference values for swine are still lacking (19, 20). This study addresses this gap by providing an accurate and reproducible imaging protocol for SE in anesthetized pigs, along with detailed reference values for cardiac dimensions and myocardial function.

## 2 Material and methods

### 2.1 Animal preparation

The experimental protocol was approved by the local federal authority (Landesamt für Verbraucherschutz und Ernährung Nordrhein-Westfalen, LAVE NRW, approval no. 84-02.04.2017.A300) and conducted in accordance with the principles of the 1964 Helsinki Declaration and its later amendments. Nineteen clinically healthy female swine (Deutsche Landrasse, *Sus scrofa domesticus*) were included. The mean body weight of the animals was 73.3 ± 8.4 kg, and they were approximately four months old.

Pigs were housed under standard conditions with a 12-h light-dark cycle for at least 7 days before the experiment, with unrestricted access to drinking water and twice-daily feedings. Twelve hours before the experiment, the animals were fasted except for water. Anesthesia was induced via intramuscular injection of azaperone (6 mg/kg), followed by intravenous administration of propofol (5–10 mg/kg/h) and fentanyl (5 µg/kg/h). Orotracheal intubation was performed, and mechanical ventilation was set to an inspiratory oxygen fraction of 0.3, a tidal volume of 10 ml/kg, and a positive end-expiratory pressure of 5 cmH_2_O. The respiratory rate was adjusted to maintain an end-tidal carbon dioxide concentration between 35 and 45 mmHg. Standard monitoring included electrocardiogram (ECG), pulse oximetry, invasive femoral arterial pressure and central venous pressure. Body temperature was maintained at 38.2°C ± 0.1°C with a convective heating device (Warm Touch 5200; Tyco Healthcare, Pleasanton, CA, USA). Following preparation, the animals received 200 IU/kg heparin, as required for the subsequent experimental procedures after echocardiography.

### 2.2 Echocardiography

#### 2.2.1 Surgical preparation

**Figure 1:**
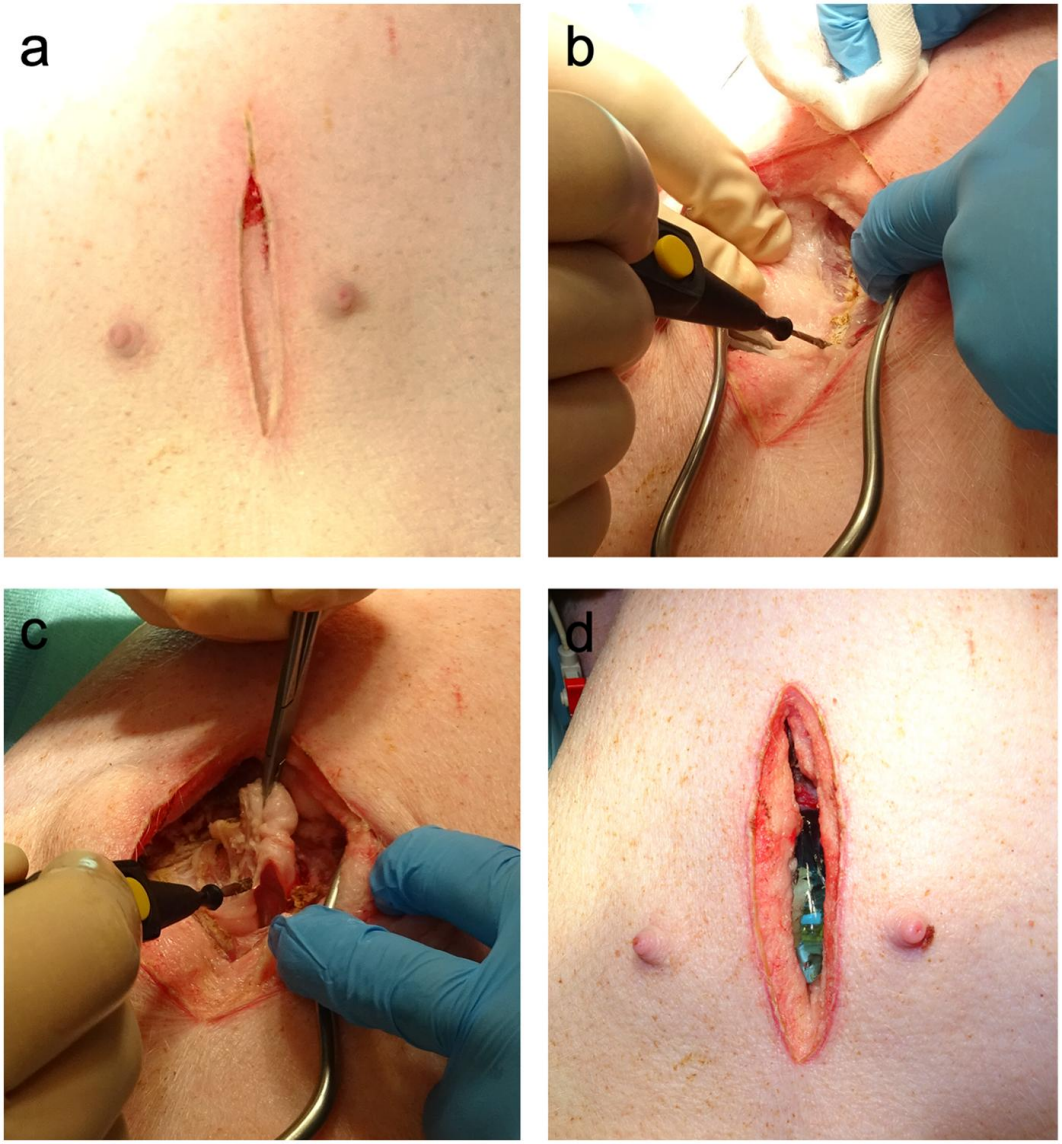
Surgical preparation for subxiphoid echocardiography. **(a)** In the first step, a median longitudinal skin incision caudal to the xiphoid was made. **(b)** The underlying connective and adipose tissues were dissected until the linea alba was visualized. Electric diathermy was used for bleeding control. **(c)** The caudal portion of the cartilaginous xiphoid was resected to gain access to the subxiphoid space. **(d)** Ultrasound gel was used to optimize the acoustic coupling of the probe with the heart.

**Figure 2:**
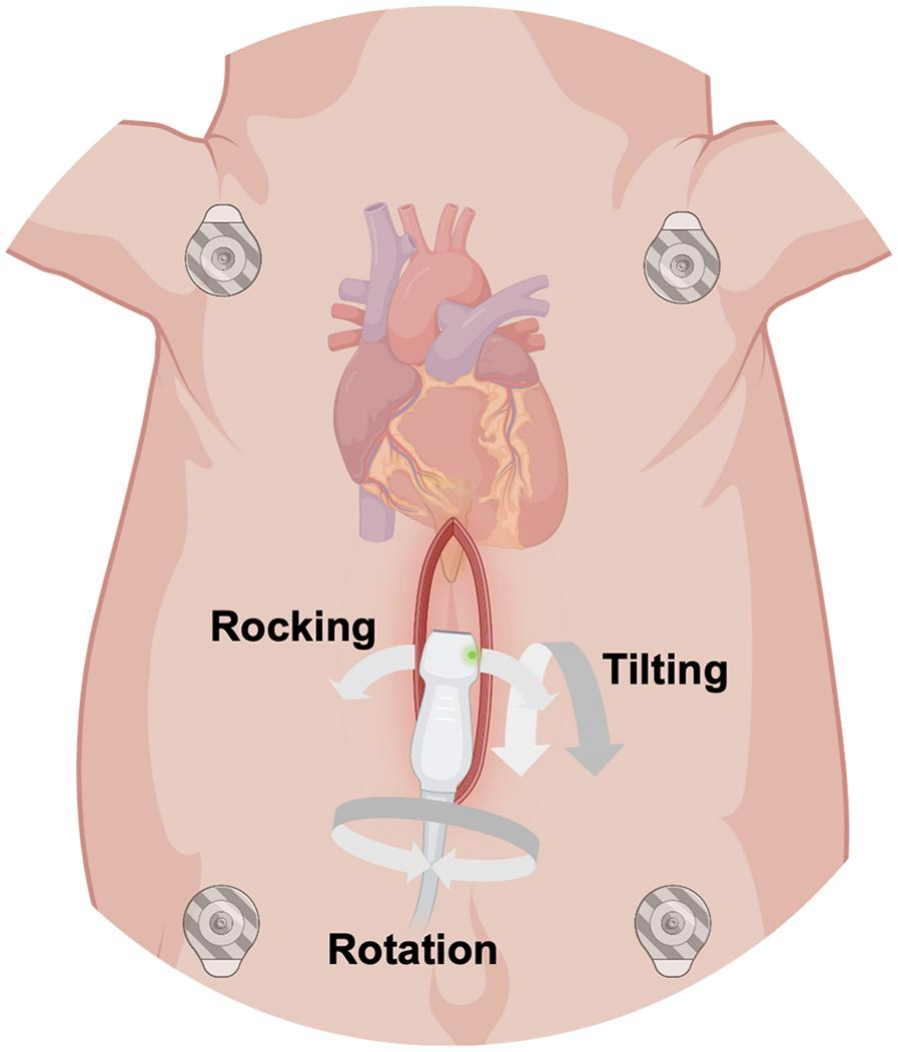
Schematic illustration of the surgical subxiphoid access to obtain epicardial apical echocardiographic views in anesthetized swine. The ultrasound probe was protected by a gel-filled probe cover and inserted into the subxiphoid space. Rotation along the longitudinal axis of the probe, tilting and rocking of the probe allowed different views of the heart. (Created with https://www.biorender.com).

The anesthetized animals were placed in dorsal recumbence. The caudal portion of the xyphoid was identified by palpation and following surgical skin preparation, a 6–8 cm midline skin incision was made caudal to the xiphoid process. Subcutaneous connective and adipose tissues were dissected until the caudal part of the xiphoid was exposed. The cartilaginous caudal portion of the xiphoid (approximately 1 cm) was resected (Figure 1). Minor tissue hemorrhage was controlled using electrical diathermy. Once the heart could be palpated, the transthoracic ultrasound probe was introduced into the subxiphoid space. To keep the wound fluids away from the transducer, the probe was protected by an ultrasound gel- filled cover. The probe was then positioned below the xiphoid in direct contact with the heart (Figure 2). If the degree of acoustic coupling was insufficient, the surgical dissection was slightly extended and ultrasound gel was applied to optimize the acoustic window. Imaging was performed by two experienced echocardiographers using a GE Vivid E9 system (GE Vingmed Ultrasound AS, Horten, Norway) equipped with a transthoracic ultrasound probe (GE Vingmed 4Vc-D, 1.4–5.2 MHz). A three-lead ECG was simultaneously recorded, and echocardiographic cine loops of three consecutive ECG-gated cardiac cycles were stored for offline analysis. Probe positioning was adjusted by both rotation along the longitudinal and axis and tilting to acquire the following standardized echocardiographic views.

#### 2.2.2 Apical 5-chamber view

**Figure 3:**
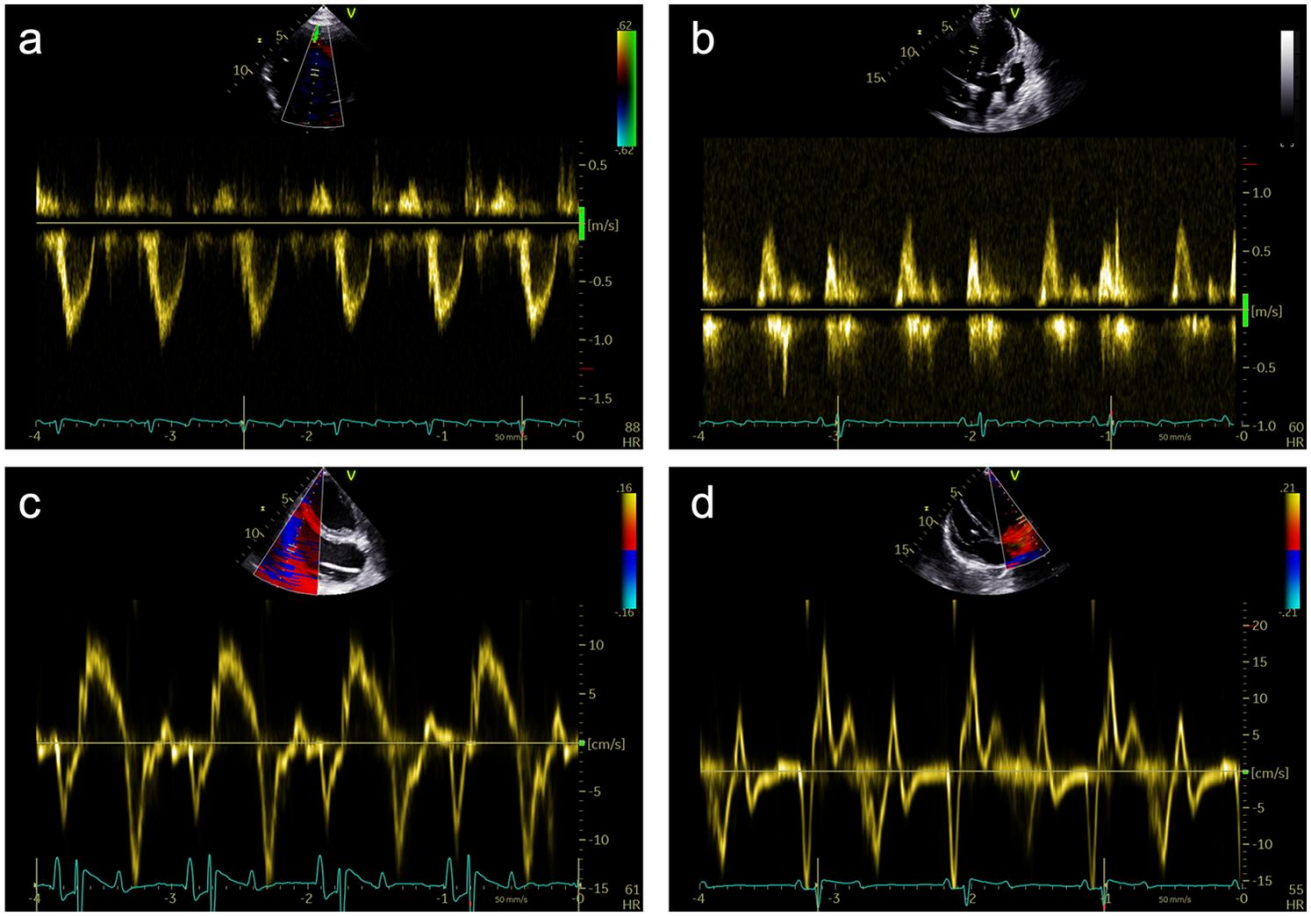
Representative pulsed-wave Doppler (PWD) and tissue Doppler (TD) measurements that were obtained during subxiphoid echocardiography of 18 female German landrace swine. **(a)** PWD measurements in the left ventricular outflow tract in an apical 5-chamber (A5C) view. **(b)** Assessment of the mitral inflow profile by PWD in an apical 3-chamber view (A3C). **(c)** Assessment of the tricuspid annular plane systolic velocity (TASV) by alignment of the TD beam with the lateral tricuspid annulus in a right ventricular focused A5C view. **(d)** Assessment of the lateral mitral annular plane diastolic velocity (E’) by alignment of the TD beam with the lateral mitral annulus in an A5C view.

The examination started with an apical 5-chamber (A5C) view. The ultrasound transducer was therefore placed subdiaphragmatically, with the probe marker oriented laterally to the left (90°–110°) and tilting slightly anteriorly (10°–20°). Due to the anatomical configuration of the swine heart, a standardized 4-chamber view is rarely obtainable. Instead, the long axis of the porcine heart extends obliquely from right to left and from posterior to anterior. This modified view allowed for clear visualization of both ventricles, although the atria could be only partially visualized (Table 1). Importantly, the left ventricular outflow tract (LVOT), the aortic valve (AV) and root can be adequately visualized in a manner analogous to a human 5-chamber view. To enhance the echocardiographic evaluation, lateral or medial adjustments of the focus improved the visualization of both ventricles, facilitating volumetric measurements and speckle-tracking analysis. Additionally, proper alignment of the Doppler beam enabled valid Doppler echocardiographic assessment of the AV and the LVOT (Figure 3).

**Table 1:** Standard two-dimensional imaging protocol for swine.

Anatomic image	2D image	Acquisition	Structures
1.1 Apical 5- chamber view-**A5C**			
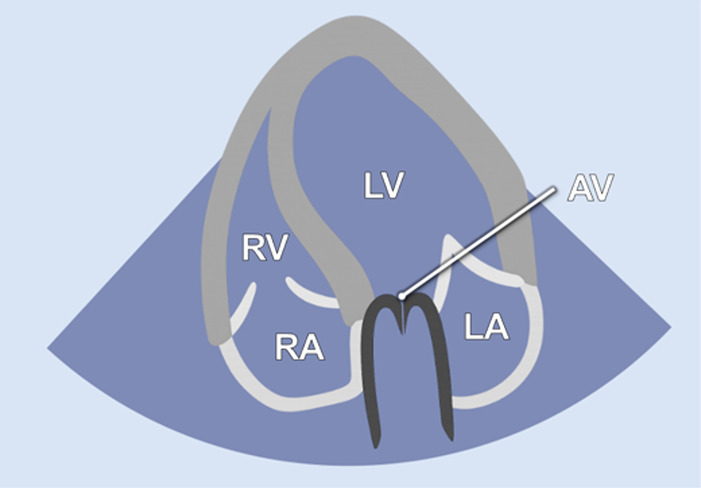	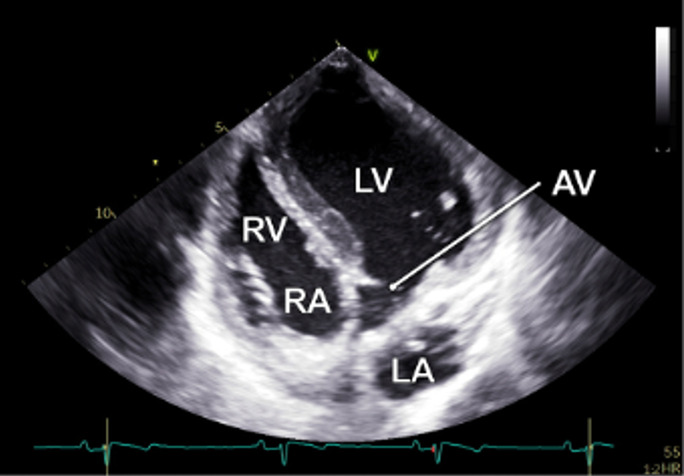	• Transducer subxiphoidal • Probe marker towards left upper limb • Focus LV	• LV • LA • AV • RV • RA
1.2 RV focused apical 5-chamber view-**A5C-RV**			
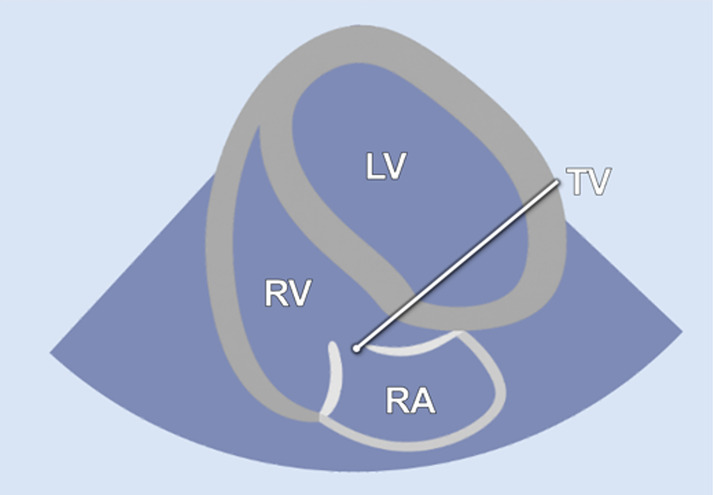	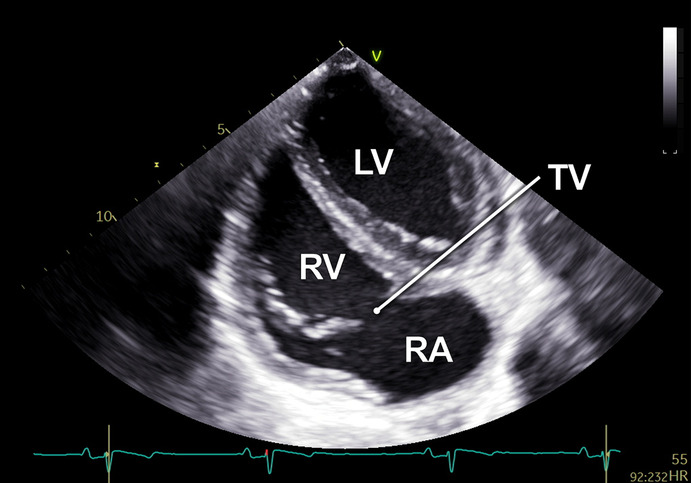	• Transducer subxiphoidal • Probe marker towards left upper limb • Focus RV (tilt anteriorly and rock medially from A5C)	• LV • RV • RA • TV
1.3 Apical 2-chamber view-**A2C**			
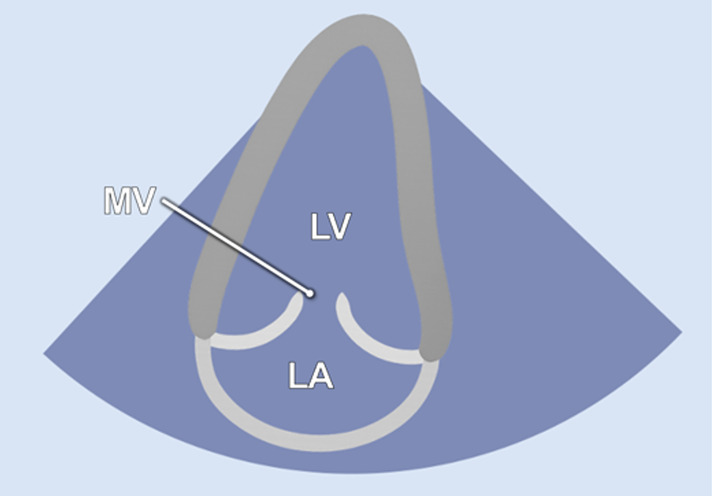	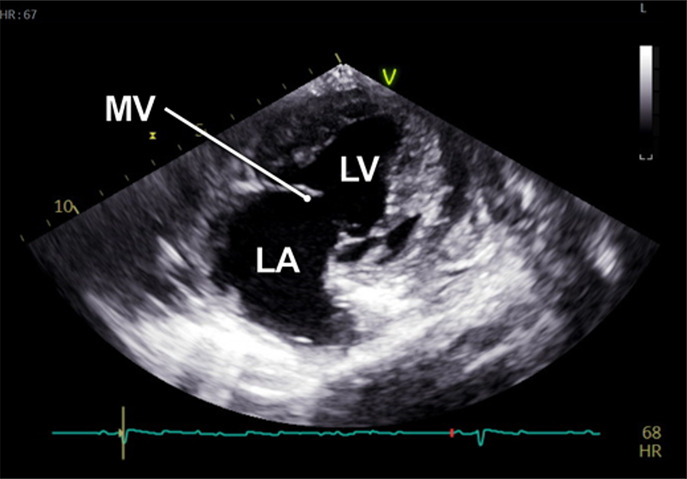	• Transducer subxiphoidal • 40°–60° counterclockwise rotation from A5C • Tilt probe posteriorly	• LV • LA • MV
1.4. Apical 3-chamber view-**A3C**			
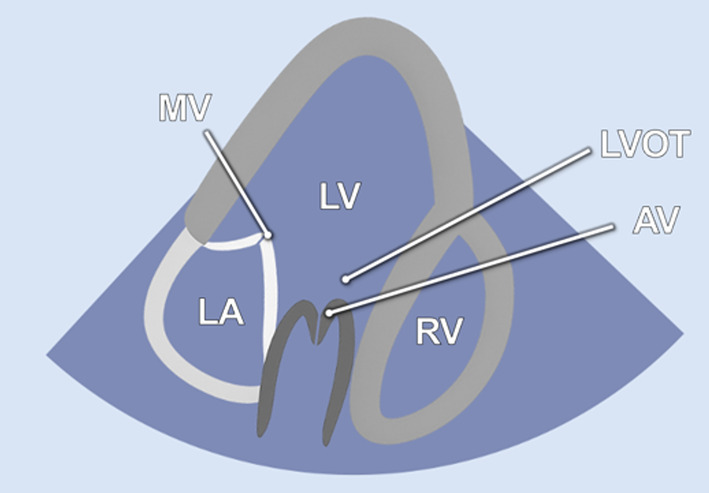	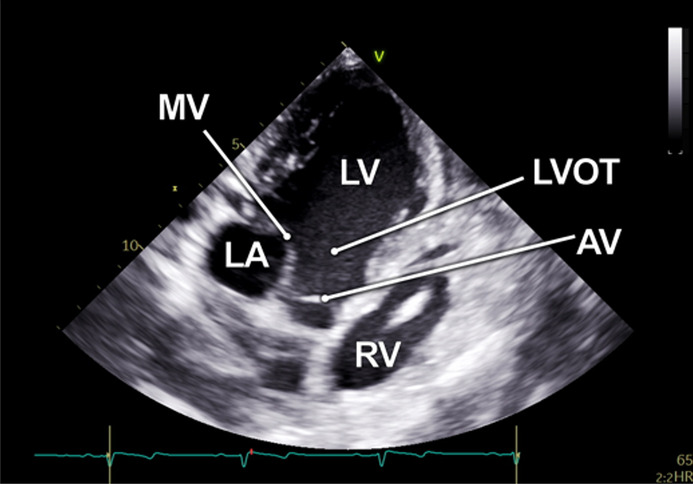	• Transducer subxiphoidal • Probe marker towards right upper limb • Tilt probe posteriorly	• LV • LA • MV • LVOT • AV • RV

#### 2.2.3 RV-focused apical 5-chamber view

In the RV-focused apical 5-chamber view (A5C-RV), the transducer was tilted anteriorly and rocked medially to optimize the visualization of the RV (Table 1). The view focuses on the RV and the tricuspid valve (TV) with partial visualization of the LV. This view was used for detailed evaluation of RV anatomy and function, including the RV free wall longitudinal strain, right ventricular basal diameter, tricuspid annular plane systolic excursion (TAPSE), and tricuspid annular systolic velocity (TASV) (Figure 3). In addition, the tricuspid valve (TV) could be assessed by Doppler echocardiography in this view.

#### 2.2.4 Apical 2-chamber view

The 2-chamber view (A2C) was obtained by rotating the transducer counterclockwise by 40°–60° from the A5C view and tilting it posteriorly. This view visualizes the LV (including the inferior and anterior wall), the mitral valve (MV) and the left atrium (LA) (Table 1). It is essential for the assessment of LV function and MV motion. Measurements of LV end-diastolic volume (LVEDV), LV end-systolic volume (LVESV), LV- global longitudinal strain (LV-GLS), Pulsed-wave (PW) Doppler and continuous-wave (CW) Doppler of the MV could be conducted in this view. Image acquisition in this view was sometimes challenging when the RV was poorly filled.

#### 2.2.5 Apical 3-chamber view

Further counterclockwise rotation of the transducer (50°–60°) and slight posterior tilting from the A2C were required to obtain the 3-chamber view (A3C). This view displays the LV (inferolateral and anteroseptal walls), the LVOT, the AV, and the MV (Table 1). It is particularly useful for imaging aortic structures and LV anatomy, allowing for the assessment of LV-GLS, as well as PW and CW Doppler of the AV.

#### 2.2.6 Inversed apical 5-chamber view

By tilting the transducer anteriorly from the A3C, the inversed 5-chamber view (iA5C) was obtained (Table 2). This view focuses on the RV and TV and partially on the LV. It served as an alternative approach for detailed assessment of the anatomy and function of the RV.

**Table 2:** Additional two-dimensional imaging protocol for swine.

Anatomic image	2D image	Acquisition	Structures
2.1 Inversed apical 5-chamber view-**iA5C**			
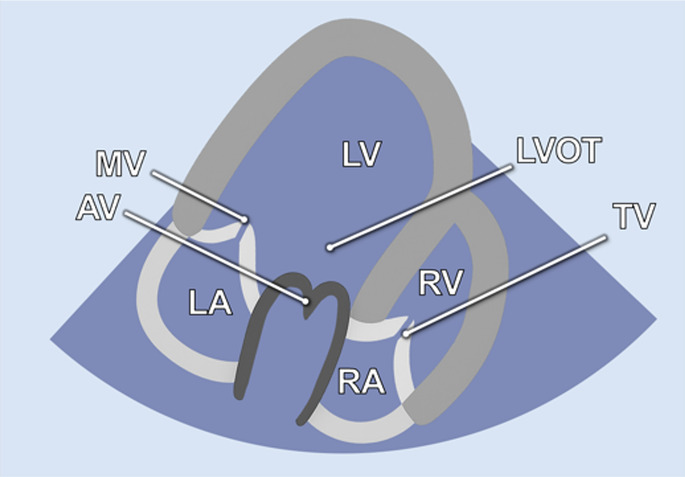	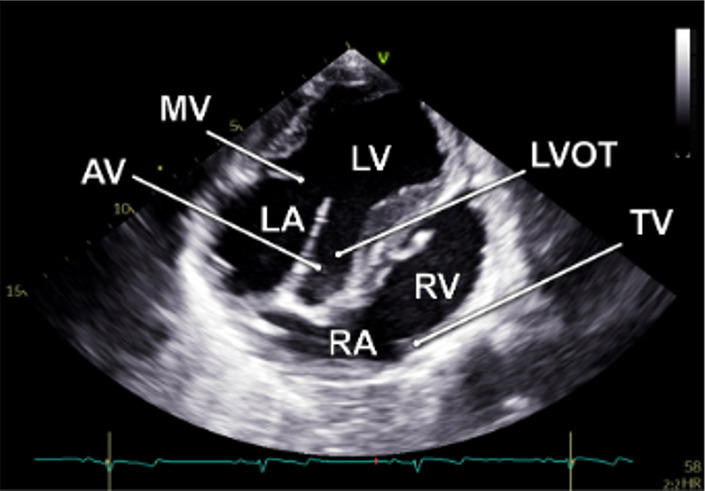	• Transducer subxiphoidal • Counterclockwise rotation from A3C • Tilt probe posteriorly	• LV • LA • MV • LVOT • AV • RV
2.2 Bicaval view- **VC**			
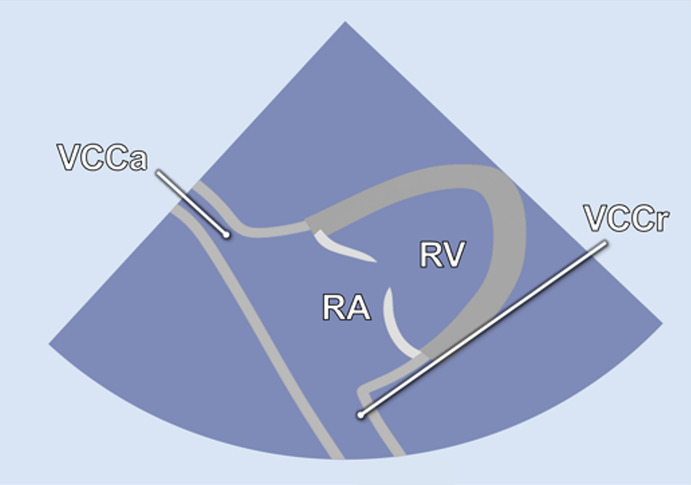	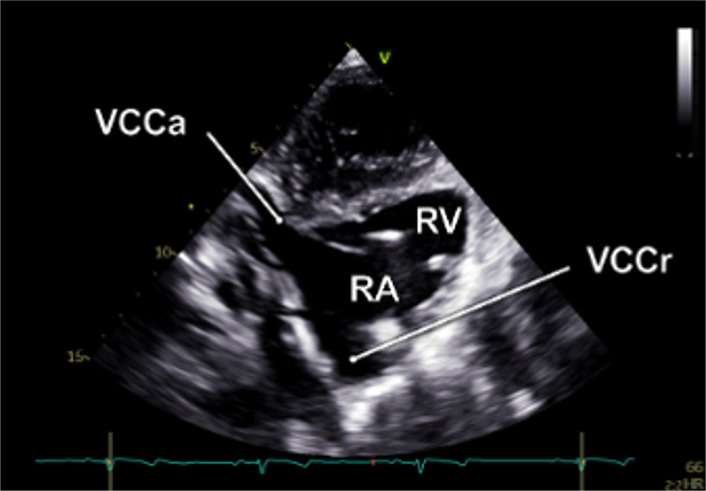	• Transducer subxiphoidal • Tilt probe anterior from iA5C	• RA • RV • VCCr • VCCa
2.3 Parasternal long Axis-P**LAX**			
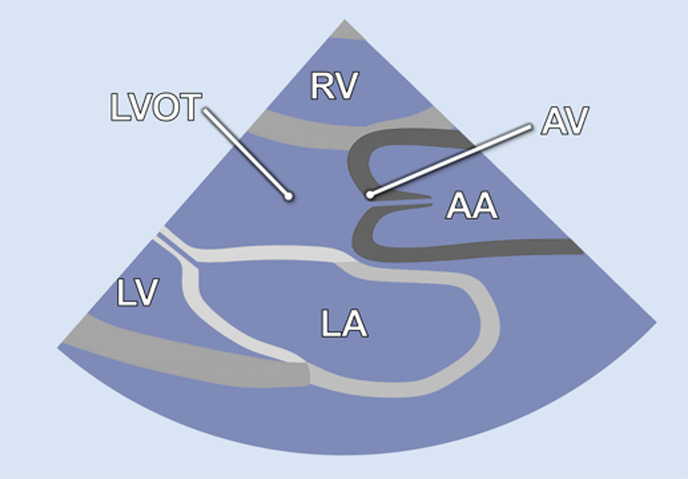	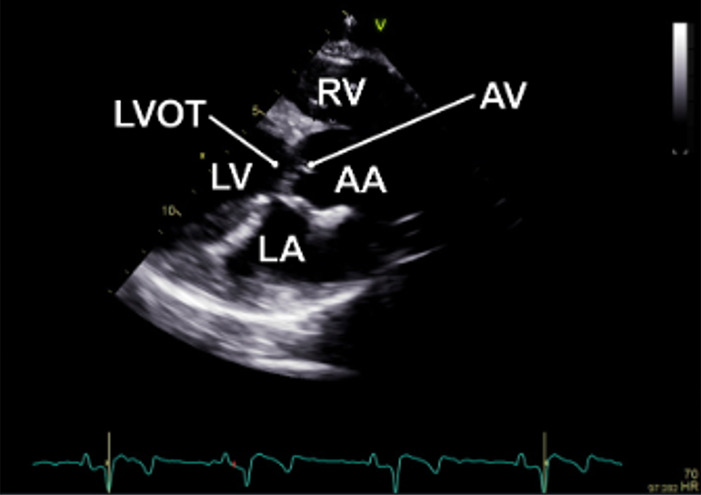	• Transducer left parasternal position	• LV • LA • LVOT • AV • RV

#### 2.2.7 Bicaval view

The bicaval view (VC) was acquired by anterior tilting of the transducer from the iA5C until the right atrium entered the imaging plane. In this view, the cranial (superior) and caudal (inferior) venae cavae (Table 2) are well visualized, whereas the LV and RV appear only marginally. The VC was primarily applied to guide catheter-based procedures.

#### 2.2.8 Parasternal long axis

The parasternal long-axis view (PLAX) was specifically utilized to measure the LVOT diameter 1–2 mm proximal to the AV leaflets during mid-systole. Therefore, the transducer was placed in the left parasternal position and the LVOT was focused (Table 2).

### 2.3 Data and image analysis

Femoral arterial and central venous pressure values were continuously monitored. The values reported in this study reflect the hemodynamic state immediately preceding the echocardiographic image acquisition.

Echocardiographic loops were analyzed using the EchoPAC suite (Version 202, GE Vingmed).

Body surface area (BSA) was calculated from the animal's body weight according to the formula by Kelley et al. (21):

$$\mathrm{BSA}\;=\;0.0734\;\times \;\mathrm{body}\;\mathrm{weigh}t^{0.656}$$

The following image analysis was performed:

LV-EF was calculated using Simpson's biplane method, combining volumetric data from the A2C and A5C views. Stroke volume (SV_VOL_) and cardiac output (CO_VOL_) were derived from these volumetric measurements.

After the LVOT diameter (d_LVOT_) was measured, the cross-sectional area (CSA_LVOT_) was calculated according to the following formula:

$$\mathrm{CS}A_{\mathrm{LVOT}}\;=\;\pi \;\times \;{\left(\frac{d_{\mathrm{LVOT}}}{2}\right)}^{2}$$

Doppler-derived stroke volume (SV_PWD_) was then calculated by multiplying the CSA with the PW Doppler-measured velocity time integral of blood flow at the LVOT (VTI_LVOT_).

$$SV_{\mathrm{PDW}}\;=\;\mathrm{CS}A_{\mathrm{LVOT}}\times \mathrm{VT}I_{\mathrm{LVOT}}$$

Cardiac output (CO_PWD_) was calculated using the ECG-derived heart rate (HR):

$$CO_{\mathrm{PWD}}\;=\;SV_{\mathrm{PWD}}\times \mathrm{HR}$$

**Figure 4:**
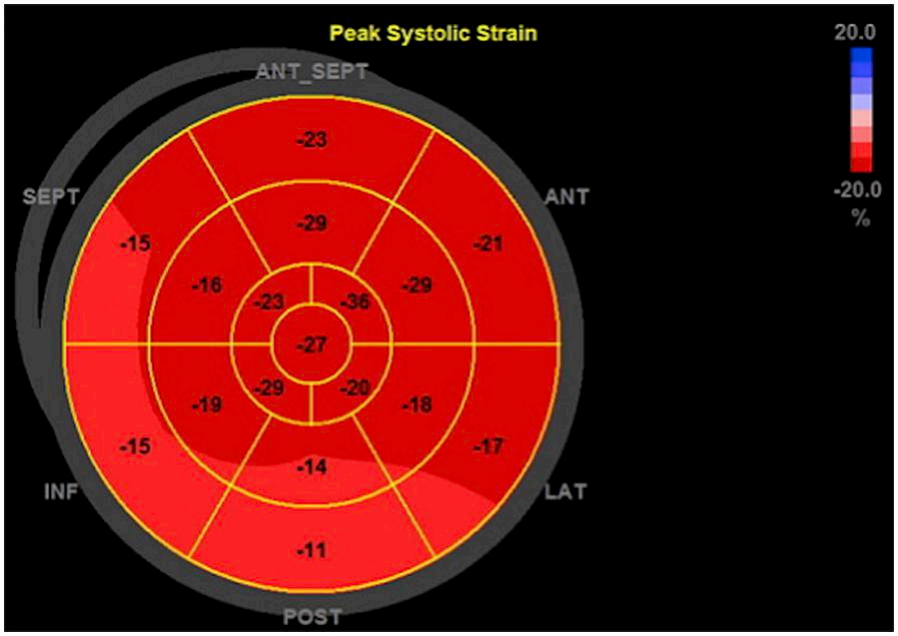
Representative bull's eye plot of the left ventricle. This figure shows a 17-segment model of the left ventricle, representing regional peak longitudinal strain values, that were determined during the myocardial deformation analysis.

Myocardial deformation analysis was performed using 2D speckle tracking. LV-GLS was calculated from the A2C, A3C and A5C views (Figure 4). The RV free wall LS was analyzed using the free wall of the right ventricle in the RVA5C view. For the RV free wall LS, the RV was segmented into six parts: the basal, mid, and apical segments of both the RV free wall and the septum. Following the manual tracing of the RV endocardial border at an end-systolic frame, an automatically generated region of interest was refined by manual adjustments of its width and position to ensure complete inclusion of the RV wall thickness. The RV free wall longitudinal strain was determined by averaging the RV lateral basal, mid, and apical segments while excluding the septal segments, as previously outlined (22, 23).

Valve function was assessed in detail. The aortic valve was evaluated in the A3C and A5C views, with measurements including mean and peak pressure gradients (MPG and PPG) and maximum velocity (AV V_max_). The mitral valve was examined in the A2C or A3C views, with early diastolic flow velocity (E), late diastolic flow velocity (A), E/A ratio, and deceleration time of the E-wave. Tissue Doppler imaging (TDI) was performed at the tricuspid annulus, septal wall and mitral annulus in the A5C view to measure regional myocardial tissue velocities.

Data processing and visualization were carried out using Microsoft Excel 2025 (Microsoft Cooperation, Redmond, WA, USA) and PRISM 10 (GraphPad Prism Version 10.2, GraphPad Software Inc., La Jolla, CA, USA). Normal distribution of the residuals was tested using the Kolmogorov–Smirnov test and diagnostic plots. Data are reported as the mean ± standard deviation (SD).

## 3 Results

A comprehensive echocardiographic examination was performed in 19 anesthetized female swine using a minimally invasive subxiphoidal surgical approach. No adverse events related to the echocardiographic examination were observed and no bleeding occurred, despite systemic anticoagulation with 200 IU/kg heparin. The procedure did not induce malignant arrythmia or hemodynamic instability. One animal demonstrated markedly elevated aortic valve velocities and pressure gradients consistent with high-grade aortic valve stenosis and was therefore excluded from the analyses. In the remaining 18 animals no valvular pathologies were observed.

### 3.1 Hemodynamics

At the time of image acquisition, all the animals were in a stable anesthetic state with sinus rhythm (Table 3). Arterial blood gas analyses (Table 3) revealed no abnormalities. The mean heart rate was 65 ± 12 beats per minute, with a mean systolic pressure of 130 ± 20 mmHg and a diastolic pressure of 66 ± 11 mmHg. The mean central venous pressure was 11 ± 1 mmHg.

**Table 3:** General, hemodynamic and blood gas data of 18 female swine.

Parameter	Value	SD
Weight [kg]	73.3	8.4
BSA [m^2^]	1.22	0.1
Heart rate [min^−1^]	65	12
Systolic arterial pressure [mmHg]	130	20
Diastolic arterial pressure [mmHg]	66	11
Mean arterial pressure [mmHg]	88	12
Central venous pressure [mmHg]	11	1
Hemoglobin [g/dl]	9.4	0.6
Glucose [mg/dl]	110	16
Lactate [mmol/L]	1.5	1.5
pH	7.49	0.03
p_a_O_2_ [mmHg]	153	30
p_a_CO_2_ [mmHg]	38	3

Weight, hemodynamic and blood gas values of 18 swine that received a subxiphoid echocardiographic examination. Data are presented as the mean ± standard deviation (SD).

### 3.2 Echocardiography

#### 3.2.1 Left ventricle

LV function and volume analysis using Simpson's biplane method (Table 4) revealed an EDV of 67 ± 16 ml and an ESV of 22 ± 8 ml, yielding an SV_VOL_ of 45 ± 13 ml and a CO_VOL_ of 2.9 ± 0.8 L/min. The ejection fraction (EF) was 67 ± 11%, and the LV-GLS was −22.4 ± 3.3%. Mitral inflow analysis yielded an E/A ratio of 1.7 ± 0.6, an E/e′ ratio of 7 ± 1.9, and an E-wave deceleration time of 217 ± 109 ms.

**Table 4:** Echocardiographic values of 18 female swine.

Parameter	Value	SD
Left ventricle		
Volume (Simpsons formula)		
End diastolic volume [ml]	67	16
End systolic volume [ml]	22	8
Ejection fraction [%]	67	11
Stroke volume_VOL_ [ml]	45	13
Cardiac output_VOL_ [L/min]	2.9	0.8
Global longitudinal strain [%]	−22.4	3.3
Aortic valve		
Peak pressure gradient [mmHg]	10.6	3
Mean pressure gradient [mmHg]	5.1	1.5
Maximum velocity [m/s]	1.6	0.3
LVOT Diameter [mm]	22	1
Velocity time integral [cm]	22.3	3.9
Aortic stroke volume_VTI_ [ml]	84	18
Aortic cardiac output_VTI_ [L/min]	5.6	1.4
Diastolic function		
E/A	1.7	0.6
E/e'	7	1.9
E-wave deceleration time [ms]	217	109
Right ventricle		
Right ventricle basal diameter [mm]	28	6
Tricuspid annular systolic velocity [cm/s]	11.2	2.3
Free wall longitudinal strain [%]	−23.7	5

Left and right ventricular echocardiographic parameters of 18 swine that received a subxiphoid echocardiographic examination. Data are presented as the mean ± standard deviation (SD).

#### 3.2.2 Aortic valve

Aortic valve assessment revealed a PPG of 11 ± 3 mmHg, an MPG of 5 ± 1 mmHg and a V_max_ at the valve of 1.6 ± 0.3 m/s. The parasternal measured LVOT diameter was 22 ± 1 mm, and the LVOT VTI was 22.3 ± 3.9 mm. SV derived from PW-Doppler (SV_VTI_) was 84 ± 18 ml, and the aortic CO_VTI_ was 5.6 ± 1.4 L/min.

#### 3.2.3 Right ventricle

RV function was assessed using 2D B-Mode echocardiography, TDI and speckle tracking. The basal RV diameter was 28 ± 6 mm, and the TASV was 11.2 ± 2.3 cm/s. The RV free wall longitudinal strain (LS) was −23.7% ± 5%.

## 4 Discussion

This study contributes to the standardization of preclinical echocardiography by providing a detailed protocol for the acquisition of apical views via a subxiphoid surgical approach in swine. The measurements reported here may serve as reference values for future echocardiographic assessments in translational research.

### 4.1 Advantages and disadvantages of SE

Before surgical SE is selected as a cardiac imaging technique, it is important to appreciate its specific advantages and disadvantages.

#### 4.1.1 Advantages

SE via a surgical approach is a safe imaging option in swine models, allowing the use of standard clinical TTE equipment. Compared with the echocardiographic approach by Galbas et al., who proposed a protocol for epicardial imaging in swine, SE significantly reduces the surgical impact on animals (20). The protocol established by Gabas et al. requires a median sternotomy, a procedure associated with considerable invasiveness, the need for advanced surgical expertise, and a potential influence on pulmonary and central venous pressures (24). In addition, epicardial echocardiography involves extensive pericardial and myocardial manipulation, which may provoke arrhythmias and thereby affect experimental outcomes. By avoiding a full sternotomy, SE preserves thoracic integrity, minimizes surgical trauma, and is particularly advantageous for studies requiring repeated imaging.

Another strength of this approach is the ability to generate high-quality LV and RV apical views through direct contact with the pericardium. This minimizes the influence of the chest wall and the lungs on the acoustic coupling of the probe with the heart. The generated apical views are particularly essential for Doppler-based measurements of tissue and blood flow velocities since they enable measurements under a correct insonation angle that cannot be achieved in a parasternal position (25, 26). Moreover, the resulting image quality allows for a precise delineation of the endocardium and myocardium, as well as adequate assessment of valvular structures (Tables 1, 2). Additionally, the method allows real-time imaging, enabling procedural guidance (Table 2, VC view).

#### 4.1.2 Disadvantages

Although the subxiphoid surgical approach greatly improved imaging, it does overcome all the challenges inherent to porcine cardiac anatomy. The rotation and direction of the axis of the swine heart may result in foreshortened views, resulting in an underestimation of volumetric measurements. Furthermore, the mini thoracotomy requires deep anesthesia for both surgical preparation and probe insertion, limiting its applicability in awake or lightly sedated animals. A theoretical risk of surgical bleeding or pneumothorax exists during dissection. However, no bleeding events were observed in the study on hand, although the preparation was conducted under anticoagulation with 200 IU/kg heparin. Under positive pressure ventilation, pneumothorax is unlikely to pose clinical concern.

While subxiphoid echocardiography provides excellent short-term imaging, its application in long-term trials is more challenging. For use in chronic models, meticulous wound closure, effective postoperative analgesia, and strict aseptic techniques are essential to minimize complications, such as infection and to ensure animal welfare. These considerations underscore the need for further investigation into the long-term feasibility and safety of this technique.

### 4.2 Limitations

This study has several limitations that should be considered when interpreting the results. First, only female swine were included. While this may limit generalizability, previous imaging studies, such as magnetic resonance tomographic analysis by Meissner et al., have demonstrated no significant sex-related differences in cardiac dimensions or function in swine models (27). Second, all measurements were performed under general anesthesia and positive pressure ventilation, that can affect myocardial function by altering preload, afterload, and contractility (28). Despite standardized anesthesia protocols and continuous monitoring of vital signs, functional parameters such as EF and strain remain subject to these influences. Third, the study was limited to an acute experimental setting. As such, the findings primarily reflect the functional reference values of subxiphoid echocardiography in an acute, anesthetized animal model. Potential long-term aspects, such as wound infections and postoperative wound healing, were not included in this study.

## 5 Conclusions

The reference values obtained in this study provide an important foundation for future research utilizing subxiphoid echocardiography in swine models. By systematically characterizing normal hemodynamic and functional parameters, in this study, a benchmark for comparison in experimental models of cardiovascular diseases, interventional procedures, and therapeutic interventions is defined. Given the increasing reliance on porcine models for translational research, the availability of a standardized echo protocol and reference data is essential for ensuring consistency and reproducibility across studies.

## Data Availability

The raw data supporting the conclusions of this article will be made available by the authors, without undue reservation.
